# Supplementary material for: The diagnostic potential of proteomics and machine learning in Lyme neuroborreliosis
Source: Nat Commun. 2025 Oct 27;16:9322. doi: 10.1038/s41467-025-64903-z (PMC12559183; doi:10.1038/s41467-025-64903-z)
Supplement: Supplementary file 1 — Supplementary Information [file 41467_2025_64903_MOESM1_ESM.pdf]

The Diagnostic Potential of Proteomics and Machine Learning in Lyme Neuroborreliosis

Supplementary Information

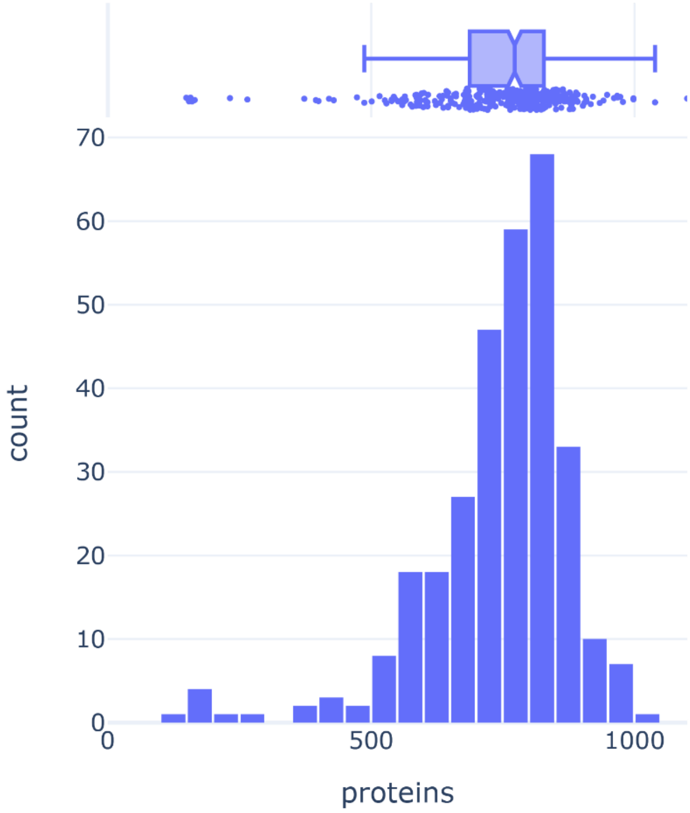

Supplementary Figure 1 The distribution of protein counts in each cerebrospinal fluid sample (n=308). Distributions represented as histogram and marginal boxplot. Samples with low protein content were removed from subsequent analysis. The cutoff for low protein count was defined by a count below the 1.5IQR from the 25th quantile in the boxplot (473 proteins).

Supplementary Table 1 Overview of proteins with significant different protein levels in cerebrospinal fluid samples of patients with Lyme neuroborreliosis (LNB) compared to viral meningitis (VM) or controls. The column "Protein.Group" represent Uniprot identifiers. The magnitude of the change is represented by log(2) fold changes in the column "log2FC". Only proteins with log2FC larger than 1 or smaller than -1 are included in the table. Log2FC is positive when the protein level is higher in LNB and negative when the protein level is higher in VM or controls. Significant proteins were identified by a two-tailed t-test adjusted for multiple hypothesis testing with Benjamini-Hochberg correction, adjusted p-values<0.05 were deemed statistically significant. Gene Ontology terms and identifiers are included in the right column.

group 1	group 2	Protein.Group	Genes	Adjusted p-value	log2FC	Gene Ontology term [indetifier]
**LNB**	VM	A0A075B6K0	IGLV3-16	2.53186E-05	1.1747	immune response [GO:0006955]
**LNB**	VM	A0A075B6K4	IGLV3-10	2.389E-07	1.8546	immune response [GO:0006955]
**LNB**	VM	A0A0B4J1Y8	IGLV9-49	2.39649E-05	1.6125	immune response [GO:0006955]
**LNB**	VM	P01591	JCHAIN	5.31028E-05	1.5848	immune response [GO:0006955]
**LNB**	VM	P01717	IGLV3-25	1.79486E-09	1.9561	immune response [GO:0006955]
**LNB**	VM	P01871	IGHM	1.9352E-05	1.7966	adaptive immune response [GO:0002250]

**LNB**	VM	P06702	S100A9	0.018078416	-1.3447	innate immune response [GO:0045087]
**LNB**	VM	P06733	ENO1	0.000228589	-1.0312	other
**LNB**	VM	P09603	CSF1	7.25579E-09	-1.2365	innate immune response [GO:0045087]
**LNB**	VM	Q9Y6R7	FCGBP	2.46493E-05	1.3237	other
**LNB**	control	A0A075B6I0	IGLV8-61	0.000309165	1.1506	immune response [GO:0006955]
**LNB**	control	A0A075B6J9	IGLV2-18	7.96928E-05	1.0590	immune response [GO:0006955]
**LNB**	control	A0A075B6K0	IGLV3-16	2.15317E-06	1.3034	immune response [GO:0006955]
**LNB**	control	A0A075B6K4	IGLV3-10	5.54358E-10	2.1034	immune response [GO:0006955]
**LNB**	control	A0A075B6P5;P01615	IGKV2-28;IGKV2D-28	3.38899E-06	1.1638	immune response [GO:0006955]
**LNB**	control	A0A0B4J1V6	IGHV3-73	6.13826E-06	1.0549	immunoglobulin mediated immune response [GO:0016064]
**LNB**	control	A0A0B4J1Y8	IGLV9-49	8.2725E-05	1.4784	immune response [GO:0006955]
**LNB**	control	A0A0C4DH24	IGKV6-21	4.49599E-07	1.1364	immune response [GO:0006955]
**LNB**	control	A0A0C4DH31	IGHV1-18	8.48379E-07	1.1144	immunoglobulin mediated immune response [GO:0016064]
**LNB**	control	A0AAG2UUW0	APOB	2.2969E-05	1.4527	other
**LNB**	control	B9A064	IGLL5	1.27217E-06	1.0648	immunoglobulin mediated immune response [GO:0016064]
**LNB**	control	O43866	CD5L	4.78052E-05	1.3132	inflammatory response [GO:0006954]
**LNB**	control	P01591	JCHAIN	4.9942E-10	2.7718	immune response [GO:0006955]
**LNB**	control	P01701	IGLV1-51	1.44117E-05	1.1102	immune response [GO:0006955]
**LNB**	control	P01709	IGLV2-8	6.36568E-05	1.1339	immune response [GO:0006955]
**LNB**	control	P01717	IGLV3-25	1.15989E-08	1.9521	immune response [GO:0006955]
**LNB**	control	P01871	IGHM	6.82279E-11	3.0667	adaptive immune response [GO:0002250]
**LNB**	control	P02741	CRP	0.00572022	-1.0117	innate immune response [GO:0045087]
**LNB**	control	P02743	APCS	3.27736E-05	1.3845	innate immune response [GO:0045087]
**LNB**	control	P02746	C1QB	4.73158E-08	1.0347	innate immune response [GO:0045087]
**LNB**	control	P04003	C4BPA	9.4724E-06	1.2708	innate immune response [GO:0045087]
**LNB**	control	P05408	SCG5	8.14822E-08	-1.1644	neuropeptide signaling pathway [GO:0007218]
**LNB**	control	P07737	PFN1	1.15996E-06	1.3794	protein stabilization [GO:0050821]
**LNB**	control	P08670	VIM	6.31901E-05	1.2797	positive regulation of gene expression [GO:0010628]
**LNB**	control	P10645	CHGA	1.49404E-07	-1.5734	innate immune response [GO:0045087]
**LNB**	control	P13796	LCP1	1.73629E-08	1.4317	cell migration [GO:0016477]
**LNB**	control	P23582	NPPC	3.57234E-09	-1.2180	angiogenesis [GO:0001525]
**LNB**	control	P26038	MSN	3.60631E-07	1.0384	positive regulation of gene expression [GO:0010628]
**LNB**	control	P31146	CORO1A	2.56422E-07	1.4438	innate immune response [GO:0045087]
**LNB**	control	P60709	ACTB	5.28812E-07	1.5228	positive regulation of cell population proliferation [GO:0008284]
**LNB**	control	P61626	LYZ	8.14426E-05	1.0573	inflammatory response [GO:0006954]
**LNB**	control	P62736;P63267	ACTA2;ACTG2	6.07352E-07	1.4786	positive regulation of gene expression [GO:0010628]
**LNB**	control	P62805	H4C1	8.61539E-06	1.8339	other
**LNB**	control	P62937	PPIA	1.83577E-05	1.0059	apoptotic process [GO:0006915]
**LNB**	control	P63104	YWHAZ	3.20261E-07	1.0842	signal transduction [GO:0007165]
**LNB**	control	Q562R1	ACTBL2	1.09819E-07	1.6610	axonogenesis [GO:0007409]
**LNB**	control	Q99574	SERPINI1	1.18025E-06	-1.2106	central nervous system development [GO:0007417]
**LNB**	control	Q9H1Z8	ECRG4	6.89432E-09	-1.0394	central nervous system development [GO:0007417]
**LNB**	control	Q9H299	SH3BGRL3	7.23881E-07	1.0751	other
**LNB**	control	Q9NZK5	ADA2	1.76646E-08	1.2921	other
**LNB**	control	Q9Y6R7	FCGBP	5.22605E-08	1.4716	other

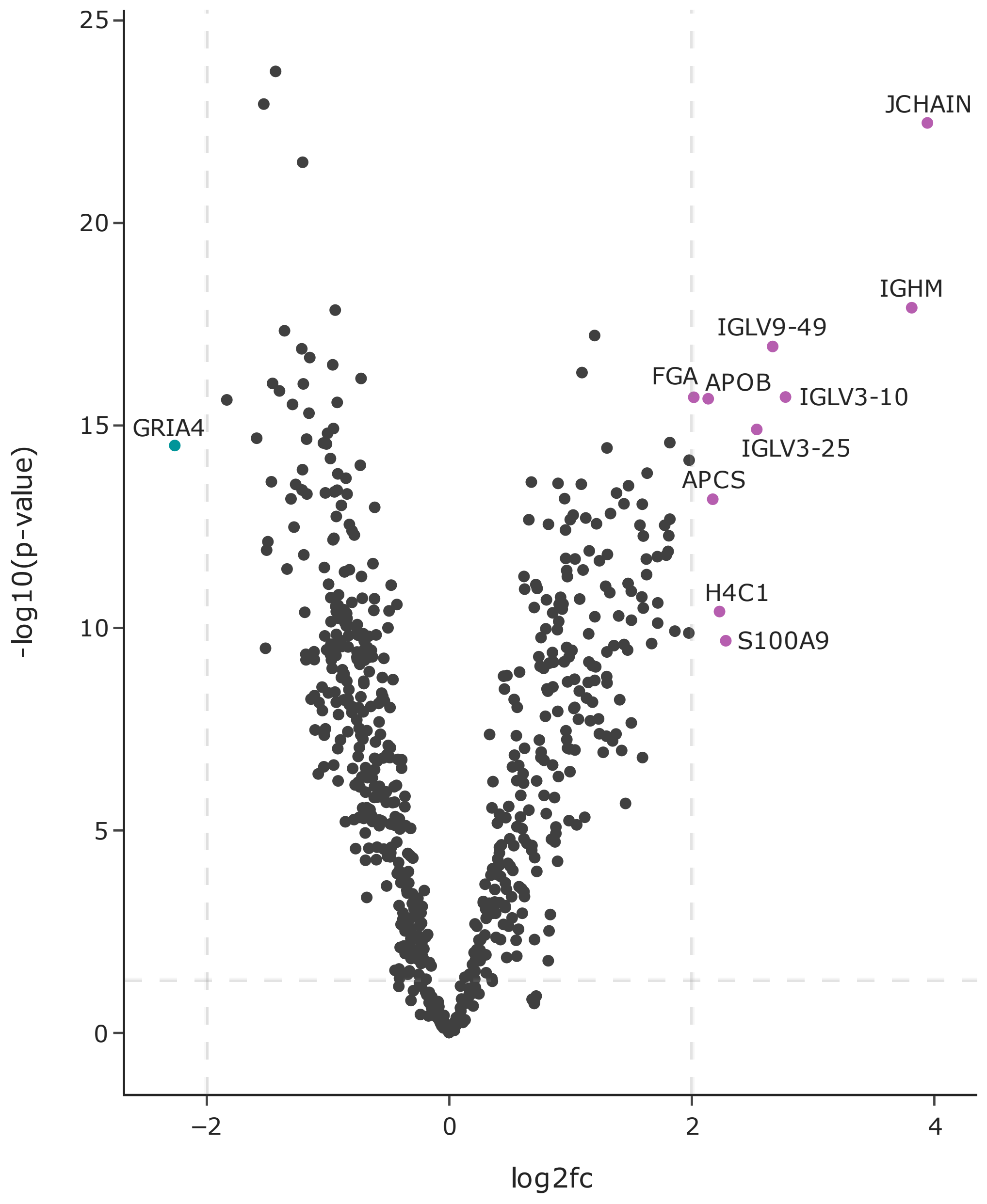

Supplementary Figure 2 Volcano plot highlighting protein differences between Lyme neuroborreliosis (LNB) and controls in the cerebrospinal fluid validation cohort. Each point represents a protein, the colour purple represents upregulated proteins with a log2 fold change (fc) larger than 2, the colour blue represents downregulated proteins with a log2 fold smaller than -2 and the colour black represents proteins with a log2 fold change between -2 and 2 or non-significant proteins. Significant proteins were identified by a two-tailed t-test adjusted for multiple hypothesis testing with Benjamini-Hochberg correction, adjusted p-values<0.05 were deemed statistically significant. X-axis depicts log2 fold change and y-axis depicts the log 10 adjusted p-value. Horizontal dashed lines represent the significance threshold after multiple hypothesis correction at p=0.05. Vertical dashed lines represent large log2 fold changes: above 2 or below -2.

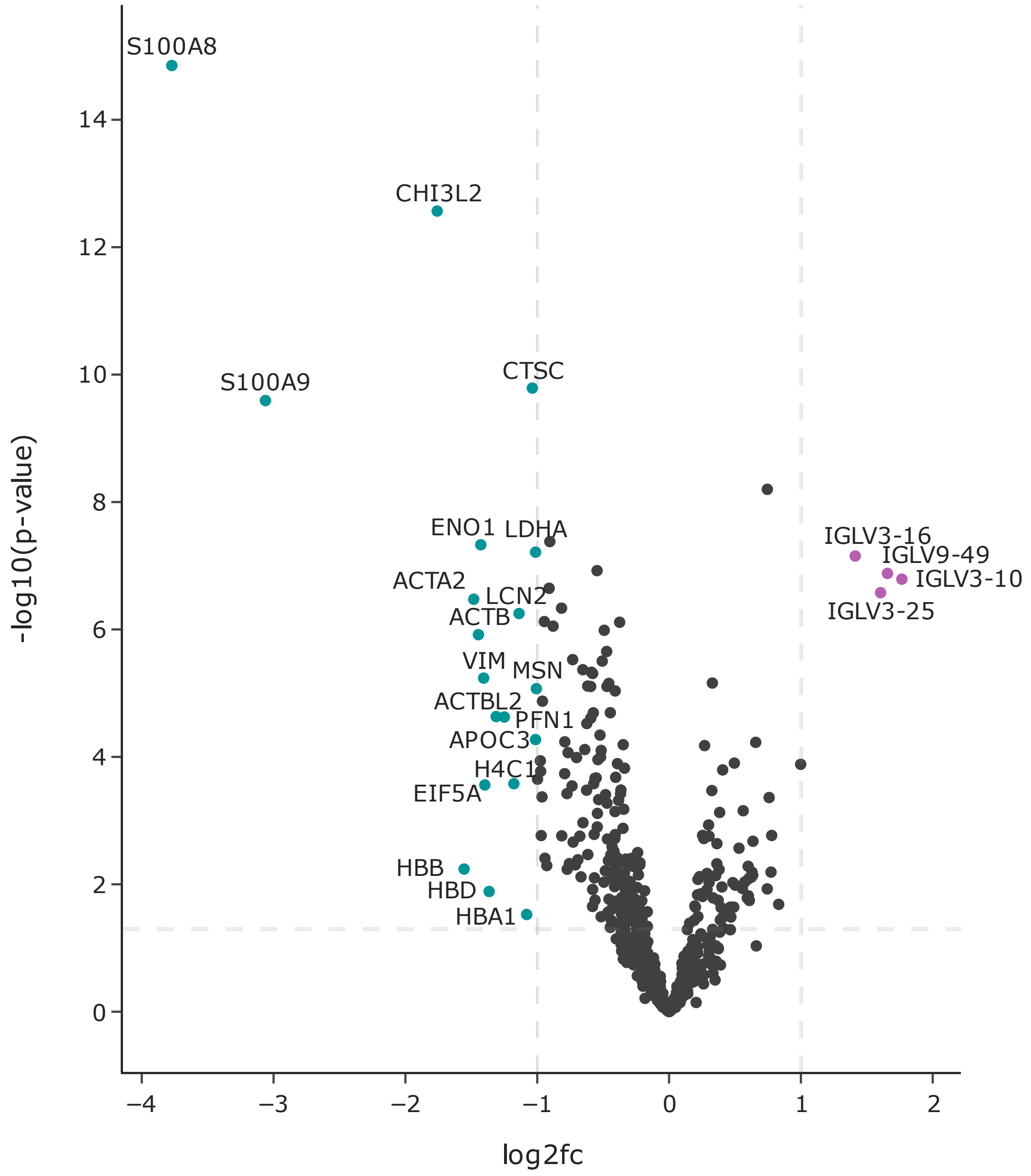

Supplementary Figure 3 Volcano plot highlighting protein differences between Lyme neuroborreliosis (LNB) and viral meningitis (VM) in the cerebrospinal fluid validation cohort. Each point represents a protein, the colour purple represents upregulated proteins with a log2 fold change (fc) larger than 1, the colour blue represents downregulated proteins with a log2 fold smaller than -1 and the colour black represents proteins with a log2 fold change between -1 and 1 or non-significant proteins. Significant proteins were identified by a two-tailed t-test adjusted for multiple hypothesis testing with Benjamini-Hochberg correction, adjusted p-values<0.05 were deemed statistically significant. X-axis depicts log2 fold change and y-axis depicts the log 10 adjusted p-value. Horizontal dashed lines represent the significance threshold after multiple hypothesis correction at p=0.05. Vertical dashed lines represent large log2 fold changes: above 1 or below -1.

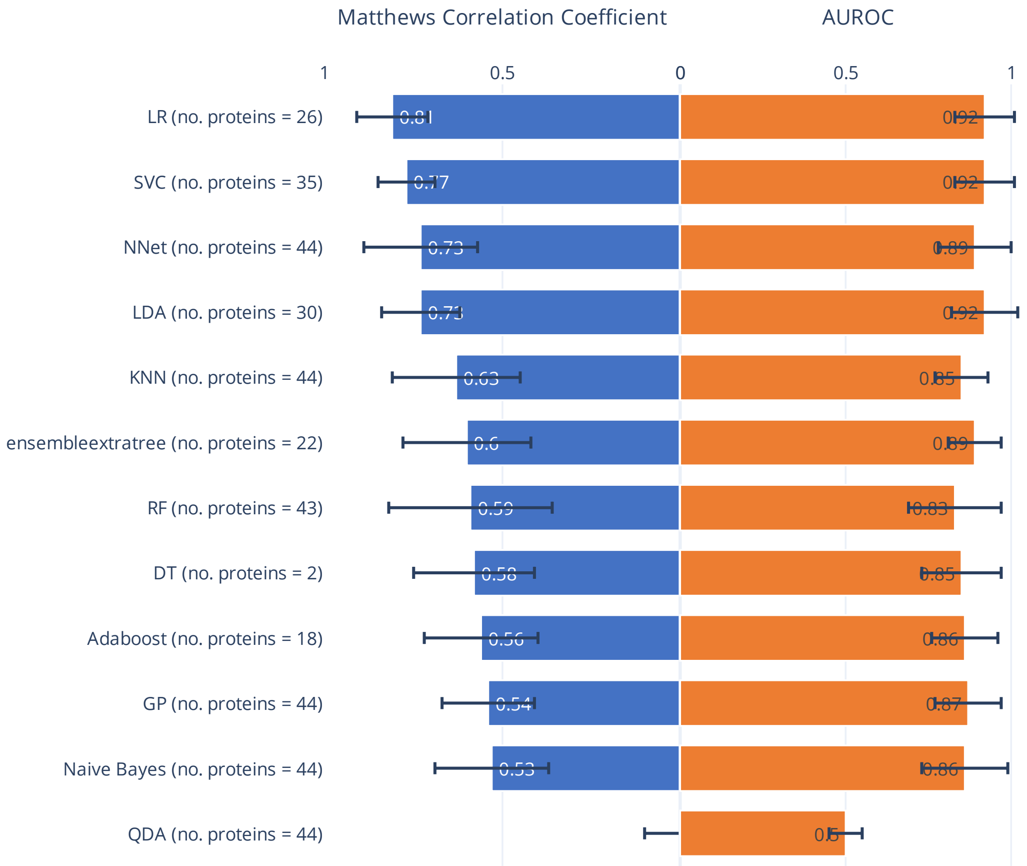

Supplementary Figure 4 Result of the cross-validation grid search of algorithms, hyperparameters, and the number of proteins is displayed as the area under the receiver operating characteristic curve (AUROC) and Matthews Correlation Coefficient (MCC) from the predictions on the test sets. The model was trained to classify Lyme neuroborreliosis and viral meningitis. The number of proteins used in each model is shown to the right. The error bars represent +/- 1 standard deviation from the mean across the 5-fold cross-validation runs.

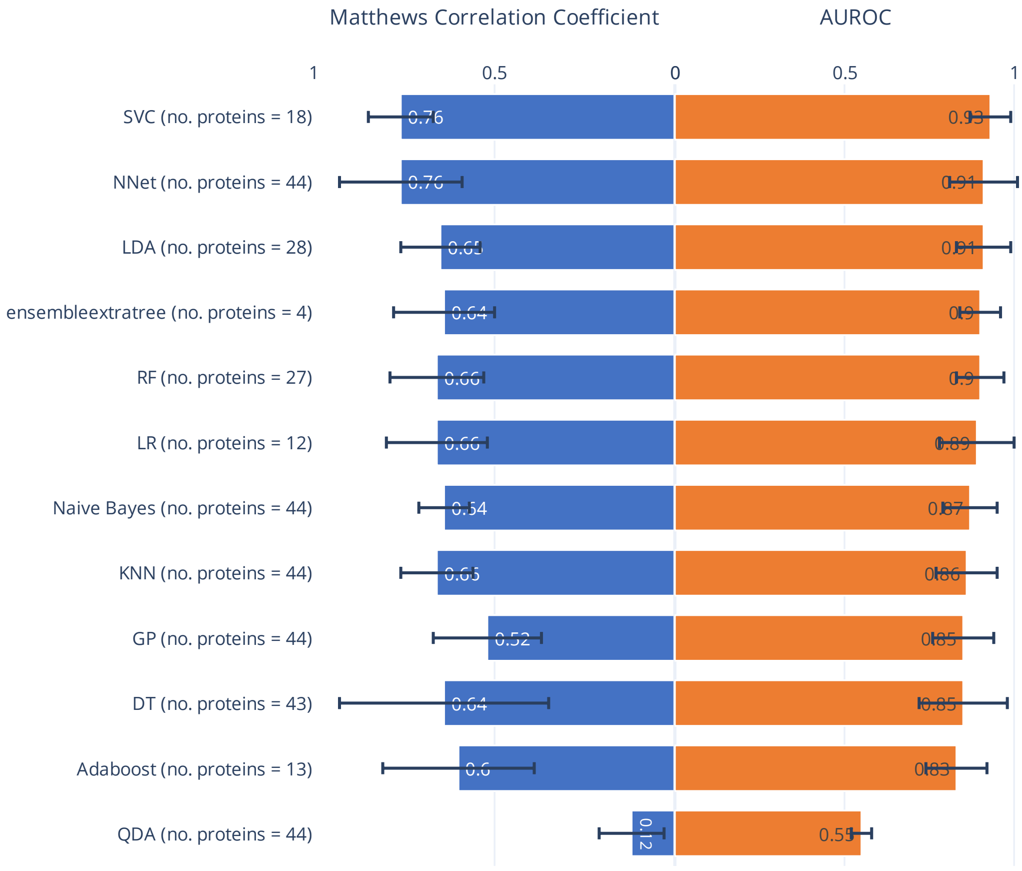

Supplementary Figure 5 Result of the cross-validation grid search of algorithms, hyperparameters, and the number of proteins is displayed as the area under the receiver operating characteristic curve (AUROC) and Matthews Correlation Coefficient (MCC) from the predictions on the test sets. The model was trained to classify Lyme neuroborreliosis and controls. The number of proteins used in each model is shown to the right. The error bars represent +/- 1 standard deviation from the mean across the 5-fold cross-validation runs.

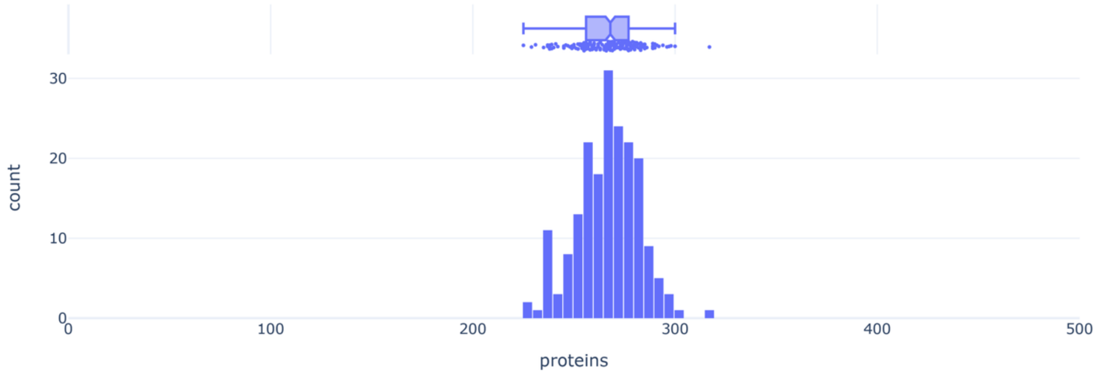

Supplementary Figure 6 The distribution of protein counts in each plasma sample (n=175)**.** Distributions represented as histogram and marginal boxplot. Samples with low protein content were removed from subsequent

analysis. The cutoff for low protein count was defined by a count below the 1.5IQR from the 25th quantile in the boxplot (230 proteins).

Supplementary Table 2 Overview of proteins with significant different protein levels in plasma samples of patients with Lyme neuroborreliosis (LNB) compared to viral meningitis (VM) or controls. The column "Protein.Group" represent Uniprot identifiers. The magnitude of the change is represented by log(2) fold changes in the column "log2FC". Log2FC is positive when the protein level is higher in LNB and negative when the protein level is higher in VM or controls. Significant proteins were identified by a two-tailed t-test adjusted for multiple hypothesis testing with Benjamini-Hochberg correction, adjusted p-values<0.05 were deemed statistically significant. Gene Ontology terms and identifiers are included in the right column.

group 1	group2	Protein.Group	Genes	Adjusted p-value	log2FC	Gene Ontology
**LNB**	VM	A0A075B6H7	IGKV3-7	0.00856	-0.35165	immune response [GO:0006955]
**LNB**	VM	A0A075B6R9;A0A0C4DH68	IGKV2-24;IGKV2D-24	0.04519	-0.39838	immune response [GO:0006955]
**LNB**	VM	A0A075B6S5	IGKV1-27	0.04927	-0.26167	immune response [GO:0006955]
**LNB**	VM	A0A0B4J1X5	IGHV3-74	0.03026	-0.30631	immunoglobulin mediated immune response [GO:0016064]
**LNB**	VM	A0A0C4DH24	IGKV6-21	0.03736	-0.38677	immune response [GO:0006955]
**LNB**	VM	A0A0C4DH31	IGHV1-18	0.01302	-0.40935	immunoglobulin mediated immune response [GO:0016064]
**LNB**	VM	A0A0C4DH36	IGHV3-38	0.01744	-0.35707	immunoglobulin mediated immune response [GO:0016064]
**LNB**	VM	A0A0J9YXX1	IGHV5-10-1	0.01870	1.13650	immunoglobulin mediated immune response [GO:0016064]
**LNB**	VM	A0AAG2TVE0	A0AAG2TVE0	0.04939	0.66621	
**LNB**	VM	O75636	FCN3	0.03694	0.19080	proteolysis [GO:0006508]
**LNB**	VM	O75882	ATRN	0.02213	0.15305	inflammatory response [GO:0006954]
**LNB**	VM	O95445	APOM	0.04778	0.18697	high-density lipoprotein particle remodeling [GO:0034375]
**LNB**	VM	P00488	F13A1	0.02867	0.24636	blood coagulation [GO:0007596]
**LNB**	VM	P01602	IGKV1-5	0.00037	-0.46912	immune response [GO:0006955]
**LNB**	VM	P01619	IGKV3-20	0.00577	-0.37231	immune response [GO:0006955]
**LNB**	VM	P01780	IGHV3-7	0.00630	-0.30620	immune response [GO:0006955]
**LNB**	VM	P01834	IGKC	0.04835	-0.25036	immune response [GO:0006955]
**LNB**	VM	P01857	IGHG1	0.00002	-0.35600	adaptive immune response [GO:0002250]
**LNB**	VM	P02649	APOE	0.02676	0.25026	cholesterol metabolic process [GO:0008203]
**LNB**	VM	P02654	APOC1	0.01812	0.25454	cholesterol metabolic process [GO:0008203]
**LNB**	VM	P02656	APOC3	0.00010	0.49565	high-density lipoprotein particle remodeling [GO:0034375]
**LNB**	VM	P02671	FGA	0.02274	-0.19636	adaptive immune response [GO:0002250]
**LNB**	VM	P02675	FGB	0.01290	-0.28546	adaptive immune response [GO:0002250]
**LNB**	VM	P02679	FGG	0.01150	-0.34442	fibrinolysis [GO:0042730]
**LNB**	VM	P02741	CRP	0.01919	-1.17647	innate immune response [GO:0045087]
**LNB**	VM	P02746	C1QB	0.04063	-0.16595	innate immune response [GO:0045087]
**LNB**	VM	P02747	C1QC	0.00153	-0.23470	immune response [GO:0006955]
**LNB**	VM	P02748	C9	0.00234	-0.37041	complement activation, classical pathway [GO:0006958]
**LNB**	VM	P02750	LRG1	0.00115	-0.49298	positive regulation of angiogenesis [GO:0045766]
**LNB**	VM	P02751	FN1	0.00439	-0.52337	acute-phase response [GO:0006953]
**LNB**	VM	P02753	RBP4	0.02769	0.23883	other
**LNB**	VM	P02765	AHSG	0.03292	-0.20536	acute-phase response [GO:0006953]
**LNB**	VM	P02790	HPX	0.00351	0.18942	intracellular iron ion homeostasis [GO:0006879]
**LNB**	VM	P03951	F11	0.04411	-0.17015	blood coagulation [GO:0007596]
**LNB**	VM	P04004	VTN	0.00231	-0.20619	immune response [GO:0006955]
**LNB**	VM	P04070	PROC	0.02816	0.20194	blood coagulation [GO:0007596]
**LNB**	VM	P04275	VWF	0.00001	-1.39211	blood coagulation [GO:0007596]
**LNB**	VM	P04433	IGKV3-11	0.03232	-0.24214	immune response [GO:0006955]
**LNB**	VM	P05090	APOD	0.00952	0.34954	angiogenesis [GO:0001525]
**LNB**	VM	P05109	S100A8	0.03735	-0.62225	innate immune response [GO:0045087]
**LNB**	VM	P05154	SERPINA5	0.00009	0.43218	lipid transport [GO:0006869]
**LNB**	VM	P05155	SERPING1	0.02323	0.17273	innate immune response [GO:0045087]
**LNB**	VM	P05546	SERPIND1	0.00690	0.21704	blood coagulation [GO:0007596]
**LNB**	VM	P06312	IGKV4-1	0.00694	-0.32065	immune response [GO:0006955]
**LNB**	VM	P06681	C2	0.00905	-0.12792	innate immune response [GO:0045087]
**LNB**	VM	P06702	S100A9	0.00011	-1.02138	innate immune response [GO:0045087]
**LNB**	VM	P06727	APOA4	0.00069	0.47736	cholesterol metabolic process [GO:0008203]
**LNB**	VM	P07195	LDHB	0.00029	0.45285	other
**LNB**	VM	P07225	PROS1	0.00001	0.25489	blood coagulation [GO:0007596]
**LNB**	VM	P07737	PFN1	0.02297	0.87449	protein stabilization [GO:0050821]
**LNB**	VM	P08185	SERPINA6	0.00647	-0.23854	other
**LNB**	VM	P08571	CD14	0.00110	-0.35763	innate immune response [GO:0045087]
**LNB**	VM	P0DJI8	SAA1	0.00290	-1.55342	acute-phase response [GO:0006953]
**LNB**	VM	P12259	F5	0.01711	-0.16784	blood coagulation [GO:0007596]
**LNB**	VM	P13473	LAMP2	0.00500	-0.29392	protein stabilization [GO:0050821]
**LNB**	VM	P13671	C6	0.00437	-0.22841	innate immune response [GO:0045087]
**LNB**	VM	P14151	SELL	0.02676	-0.27011	cell adhesion [GO:0007155]
**LNB**	VM	P20742	PZP	0.03131	-0.84446	other
**LNB**	VM	P24592	IGFBP6	0.02466	0.25876	negative regulation of cell population proliferation [GO:0008285]
**LNB**	VM	P29622	SERPINA4	0.00217	0.28261	other
**LNB**	VM	P35858	IGFALS	0.00401	-0.30860	cell adhesion [GO:0007155]
**LNB**	VM	P51884	LUM	0.00808	0.28393	other
**LNB**	VM	P60709	ACTB	0.00368	1.30850	platelet aggregation [GO:0070527]
**LNB**	VM	P80108	GPLD1	0.00196	0.26208	negative regulation of cell population proliferation [GO:0008285]
**LNB**	VM	Q16610	ECM1	0.01922	-0.22499	inflammatory response [GO:0006954]
**LNB**	VM	Q96IY4	CPB2	0.00794	0.17087	blood coagulation [GO:0007596]
**LNB**	VM	Q9NPH3	IL1RAP	0.02190	-0.38014	immune response [GO:0006955]
**LNB**	VM	Q9NZP8	C1RL	0.00146	-0.19024	innate immune response [GO:0045087]

**LNB**	control	A0A075B6H7	IGKV3-7	0.00177	-0.33554	immune response [GO:0006955]
**LNB**	control	A0A075B6P5;P01615	IGKV2-28;IGKV2D-28	0.01413	-0.29223	immune response [GO:0006955]
**LNB**	control	A0A075B6R9;A0A0C4DH68	IGKV2-24;IGKV2D-24	0.00432	-0.43494	immune response [GO:0006955]
**LNB**	control	A0A087WW87;P01614	IGKV2-40;IGKV2D-40	0.00049	-0.49442	immune response [GO:0006955]
**LNB**	control	A0A0B4J1V0	IGHV3-15	0.01137	-0.29754	immunoglobulin mediated immune response [GO:0016064]
**LNB**	control	A0A0B4J1X5	IGHV3-74	0.01247	-0.31114	immunoglobulin mediated immune response [GO:0016064]
**LNB**	control	A0A0C4DH25	IGKV3D-20	0.00329	-0.44289	immune response [GO:0006955]
**LNB**	control	A0A0C4DH33	IGHV1-24	0.01431	-0.45928	immunoglobulin mediated immune response [GO:0016064]
**LNB**	control	A0A0C4DH36	IGHV3-38	0.00167	-0.44729	immunoglobulin mediated immune response [GO:0016064]
**LNB**	control	A0A0J9YX35	IGHV3-64D	0.01266	-0.24660	immunoglobulin mediated immune response [GO:0016064]
**LNB**	control	A0M8Q6	IGLC7	0.02357	-0.18057	adaptive immune response [GO:0002250]
**LNB**	control	O75636	FCN3	0.00312	0.23870	proteolysis [GO:0006508]
**LNB**	control	P00488	F13A1	0.00791	0.21285	blood coagulation [GO:0007596]
**LNB**	control	P00734	F2	0.02876	-0.10192	blood coagulation [GO:0007596]
**LNB**	control	P00738	HP	0.01893	0.38625	acute-phase response [GO:0006953]
**LNB**	control	P00740	F9	0.04619	0.09905	blood coagulation [GO:0007596]
**LNB**	control	P00747	PLG	0.00278	-0.16783	blood coagulation [GO:0007596]
**LNB**	control	P01011	SERPINA3	0.01873	0.15279	acute-phase response [GO:0006953]
**LNB**	control	P01024	C3	0.00184	0.16474	immune response [GO:0006955]
**LNB**	control	P01031	C5	0.00135	0.18593	complement activation, classical pathway [GO:0006958]
**LNB**	control	P01042	KNG1	0.00865	-0.10983	blood coagulation [GO:0007596]
**LNB**	control	P01619	IGKV3-20	0.00009	-0.40547	immune response [GO:0006955]
**LNB**	control	P01780	IGHV3-7	0.01094	-0.30028	immune response [GO:0006955]
**LNB**	control	P01834	IGKC	0.00367	-0.30026	immune response [GO:0006955]
**LNB**	control	P01857	IGHG1	0.00007	-0.32923	adaptive immune response [GO:0002250]
**LNB**	control	P01861	IGHG4	0.03574	-0.45341	adaptive immune response [GO:0002250]
**LNB**	control	P02671	FGA	0.03067	-0.13049	adaptive immune response [GO:0002250]
**LNB**	control	P02743	APCS	0.03289	0.20412	innate immune response [GO:0045087]
**LNB**	control	P02746	C1QB	0.02157	-0.11934	innate immune response [GO:0045087]
**LNB**	control	P02747	C1QC	0.00066	-0.21374	immune response [GO:0006955]
**LNB**	control	P02751	FN1	0.00001	-0.63677	acute-phase response [GO:0006953]
**LNB**	control	P02765	AHSG	0.00000	-0.40582	acute-phase response [GO:0006953]
**LNB**	control	P02766	TTR	0.02683	-0.42209	other
**LNB**	control	P02787	TF	0.01237	-0.16740	antibacterial humoral response [GO:0019731]
**LNB**	control	P02790	HPX	0.00311	0.15382	intracellular iron ion homeostasis [GO:0006879]
**LNB**	control	P03951	F11	0.04714	-0.12687	blood coagulation [GO:0007596]
**LNB**	control	P03952	KLKB1	0.00873	-0.14870	blood coagulation [GO:0007596]
**LNB**	control	P04003	C4BPA	0.04015	0.11068	innate immune response [GO:0045087]
**LNB**	control	P04180	LCAT	0.00839	-0.14726	cholesterol metabolic process [GO:0008203]
**LNB**	control	P04196	HRG	0.02380	-0.16257	fibrinolysis [GO:0042730]
**LNB**	control	P04275	VWF	0.00001	-1.15025	blood coagulation [GO:0007596]
**LNB**	control	P04430	IGKV1-16	0.04914	-0.21898	immune response [GO:0006955]
**LNB**	control	P04433	IGKV3-11	0.00013	-0.35888	immune response [GO:0006955]
**LNB**	control	P05109	S100A8	0.00013	-0.99785	innate immune response [GO:0045087]
**LNB**	control	P05154	SERPINA5	0.04019	0.21592	lipid transport [GO:0006869]
**LNB**	control	P05155	SERPING1	0.00778	0.24329	innate immune response [GO:0045087]
**LNB**	control	P05452	CLEC3B	0.01633	-0.19006	ossification [GO:0001503]
**LNB**	control	P05546	SERPIND1	0.00841	0.16932	blood coagulation [GO:0007596]
**LNB**	control	P06312	IGKV4-1	0.00054	-0.30975	immune response [GO:0006955]
**LNB**	control	P06396	GSN	0.00687	-0.18286	positive regulation of gene expression [GO:0010628]
**LNB**	control	P06702	S100A9	0.00002	-1.00485	innate immune response [GO:0045087]
**LNB**	control	P07195	LDHB	0.00565	0.29196	other
**LNB**	control	P07225	PROS1	0.00067	0.14975	blood coagulation [GO:0007596]
**LNB**	control	P07737	PFN1	0.04692	0.72079	protein stabilization [GO:0050821]
**LNB**	control	P07996	THBS1	0.01845	-0.45506	immune response [GO:0006955]
**LNB**	control	P08185	SERPINA6	0.01638	-0.17548	other
**LNB**	control	P0C0L4	C4A	0.01764	0.21733	innate immune response [GO:0045087]
**LNB**	control	P0DOY2;P0DOY3	IGLC2;IGLC3	0.00386	-0.43963	adaptive immune response [GO:0002250]
**LNB**	control	P13473	LAMP2	0.00760	-0.22657	protein stabilization [GO:0050821]
**LNB**	control	P14151	SELL	0.03272	-0.20318	cell adhesion [GO:0007155]
**LNB**	control	P20742	PZP	0.02527	-0.55127	other
**LNB**	control	P35858	IGFALS	0.00310	-0.23778	cell adhesion [GO:0007155]
**LNB**	control	P60709	ACTB	0.02817	0.52901	platelet aggregation [GO:0070527]
**LNB**	control	P61769	B2M	0.01854	0.24553	positive regulation of immune response [GO:0050778]
**LNB**	control	Q14624	ITIH4	0.00441	0.11570	acute-phase response [GO:0006953]
**LNB**	control	Q15582	TGFBI	0.00302	-0.34742	cell adhesion [GO:0007155]
**LNB**	control	Q16610	ECM1	0.00008	-0.32219	inflammatory response [GO:0006954]
**LNB**	control	Q96IY4	CPB2	0.00369	0.18317	blood coagulation [GO:0007596]
**LNB**	control	Q9UGM5	FETUB	0.02573	-0.25847	other

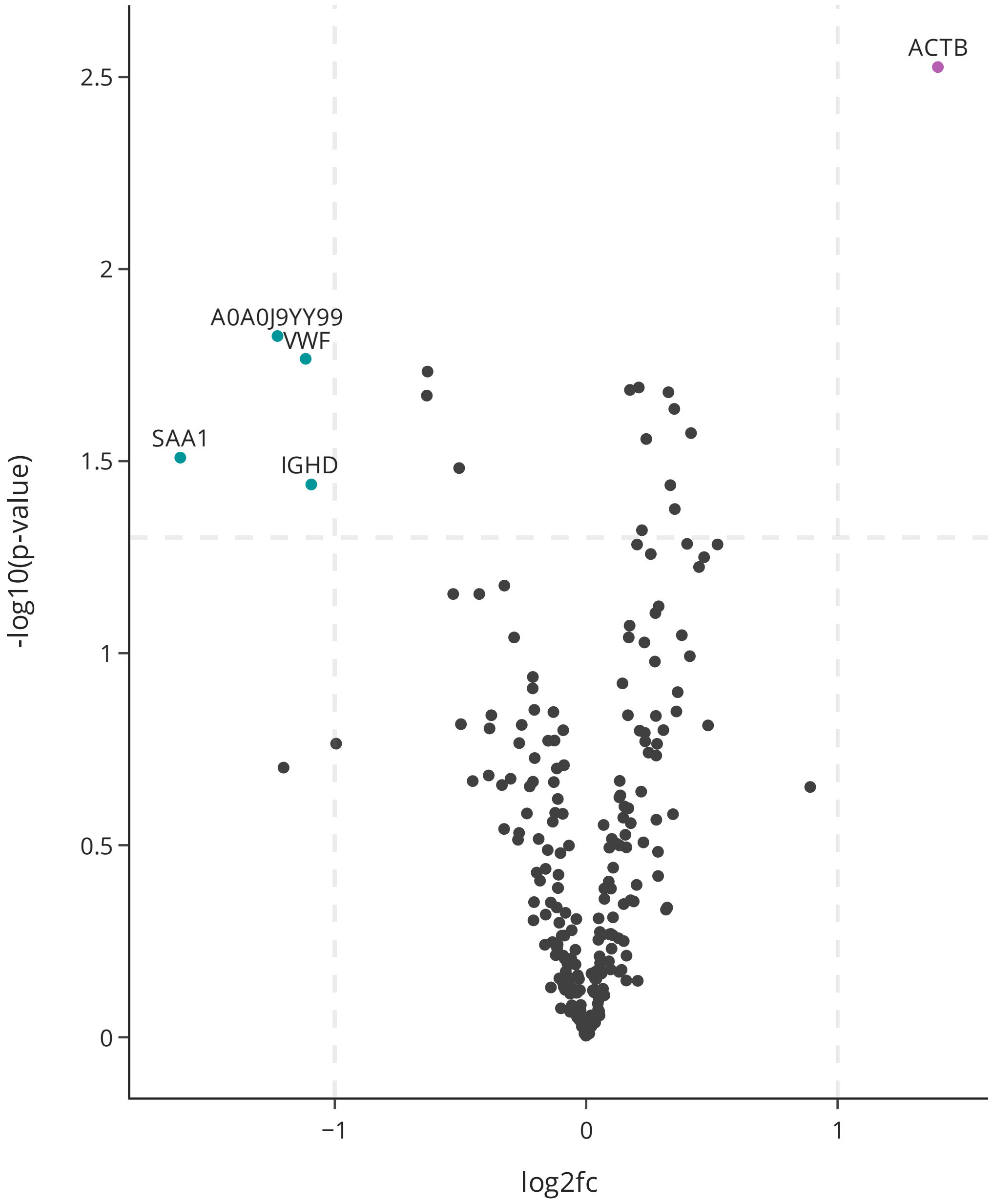

Supplementary Figure 7 Volcano plot highlighting protein differences between Lyme neuroborreliosis (LNB) and controls in the plasma validation cohort. Each point represents a protein, the colour purple represents upregulated proteins with a log2 fold change (FC) larger than 1, the colour blue represents downregulated proteins with a log2 fold smaller than -1 and the colour black represents proteins with a log2 fold change between -1 and 1 or non-significant proteins. Significant proteins were identified by a two-tailed t-test adjusted for multiple hypothesis testing with Benjamini-Hochberg correction, adjusted p-values<0.05 were deemed statistically significant. X-axis depicts log2 fold change and y-axis depicts the log 10 adjusted p-value. Horizontal dashed lines represent the significance threshold after multiple hypothesis correction at p=0.05. Vertical dashed lines represent large log2 fold changes: above 1 or below -1.

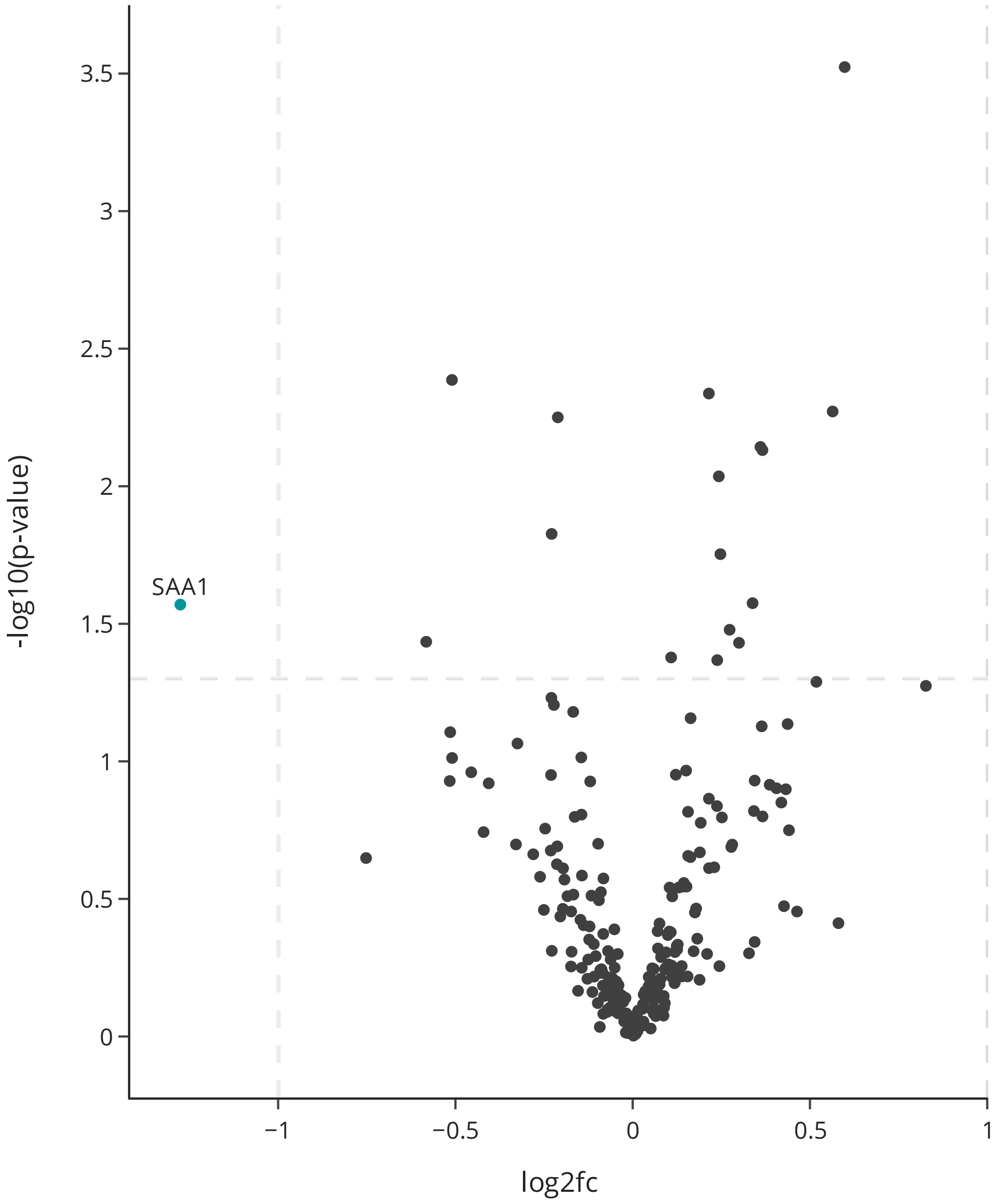

Supplementary Figure 8 Volcano plot highlighting protein differences between Lyme neuroborreliosis (LNB) and viral meningitis (VM) the plasma validation cohort. Each point represents a protein, the colour purple represents upregulated proteins with a log2 fold change (FC) larger than 1, the colour blue represents downregulated proteins with a log2 fold smaller than -1 and the colour black represents proteins with a log2 fold change between -1 and 1 or non-significant proteins. Significant proteins were identified by a two-tailed t-test adjusted for multiple hypothesis testing with Benjamini-Hochberg correction, adjusted p-values<0.05 were deemed statistically significant. X-axis depicts log2 fold change and y-axis depicts the log 10 adjusted p-value. Horizontal dashed lines represent the significance threshold after multiple hypothesis correction at p=0.05. Vertical dashed lines represent large log2 fold changes: above 1 or below -1.

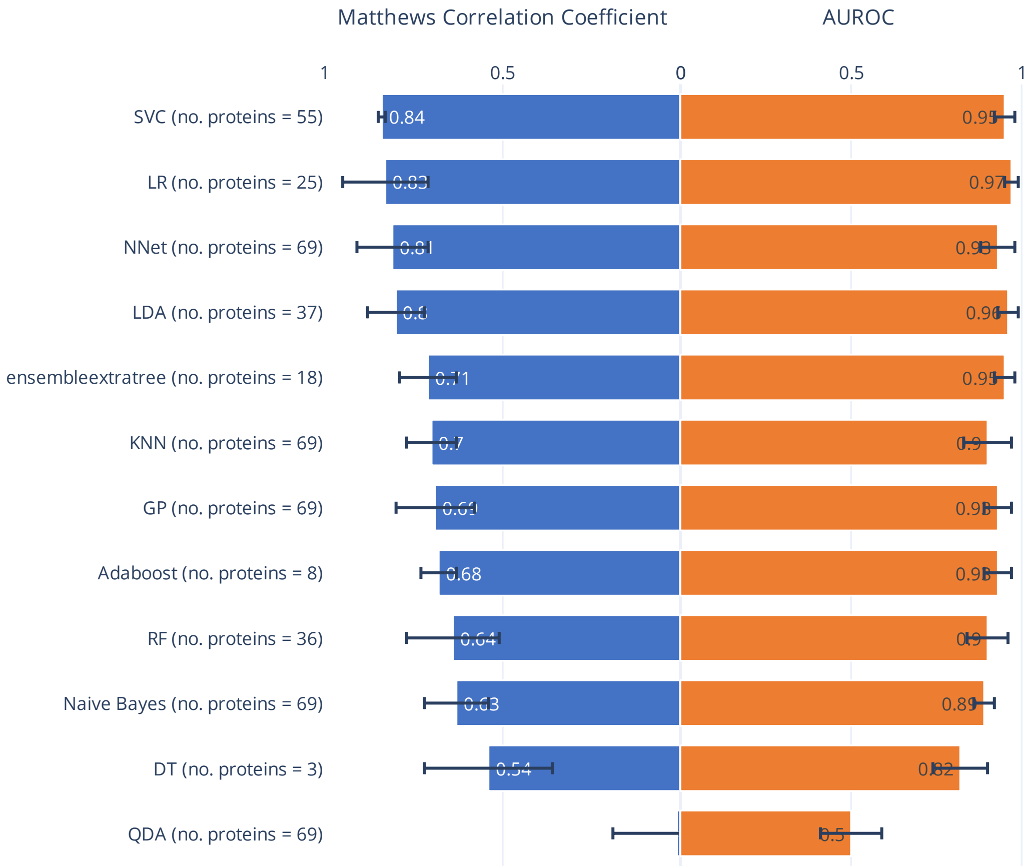

Supplementary Figure 9 Result of the cross-validation grid search of algorithms, hyperparameters, and the number of proteins is displayed as the area under the receiver operating characteristic curve (AUROC) and Matthews Correlation Coefficient (MCC) from the predictions on the test sets. The model was trained to classify Lyme neuroborreliosis and controls. The number of proteins used in each model is shown to the right. The error bars represent +/- 1 standard deviation from the mean across the 3-fold cross-validation runs.

Supplementary Table 3 Overlap of significant proteins from the comparison of Lyme neuroborreliosis (LNB) to viral meningitis (VM) or controls in cerebrospinal fluid (CSF) and plasma. Significant differences in protein levels are represented by log(2) fold change. Non-signigicant findings are not included (missing values). Significant proteins were identified by a two-tailed t-test adjusted for multiple hypothesis testing with Benjamini-Hochberg correction, adjusted p-values<0.05 were deemed statistically significant.

	**LNB compared to:**			
**Gene Names**	VM (CSF)	Control (CSF)	VM (plasma)	Control (plasma)
VTN	0.03486			-0.04154
HRG	0.10060		0.07463	
IGKV2-28			-0.29411	
CPB2	0.12280			
CLEC3B	-0.05174		0.10654	
C6	-0.13418			-0.07783
PROC		-0.19327		0.06601
F2	-0.00860		-0.01883	
CD14	0.12670			-0.12986
SELL	0.04149			
AHSG	-0.19228			
SERPINA4	0.18069			0.00656
IGLC7			-0.04297	
TTR	0.03729		-0.06505	
S100A9		0.84752		
APOC3		-0.00084		0.15146
GPLD1	0.03883			-0.06731
IGKV1-5				-0.21853
FETUB	0.03901		-0.16283	
APOC1	-0.05587			0.08117

ITIH4	0.10830		0.02824	
FGG	0.15930			-0.12332
C9	0.17234			0.12583
SERPINA5	-0.23208			
KNG1	-0.00931		-0.03355	
IGFALS	0.09572			
HPX	0.04750			
IGHV3-38	0.29536			
C2	0.08790			0.02378
ATRN				-0.06195
IGKV1-27	0.10777			-0.15922
KLKB1	0.10671		0.01229	
IGHV1-18				-0.13717
APCS	-0.15666		-0.00795	
CRP				0.06933
IGHV3-15			-0.22683	
LUM				0.01354
IGKV2-40	0.36117		-0.20312	
C4BPA	0.33873		0.11014	
APOD				0.18827
C3			0.04487	
S100A8	-1.08517			
IGHG4	0.20368		-0.10122	
FGB	0.19970			-0.06845
APOE	0.11455			0.14129
RBP4	0.18832			0.12787
IGFBP6	-0.21004			0.13004
IGKV1-16	0.44449		-0.15719	
APOA4	-0.04396			0.05237
APOM	0.11486			-0.07277
ACTB	-0.16814			
B2M	0.11084		0.28193	
IGKV3D-20			-0.30725	
PLG	-0.04959		-0.07125	
C1QC	0.28355			
C5	0.28369		0.05656	
C1RL	-0.04385			-0.04555
FGA	-0.04725			
PFN1	-0.41513			
IGKV6-21				-0.16605

Supplementary Table 4 Overlap of significant proteins from the current study with significant proteins from other LNB proteome studies. Note that the list does not cover all identified proteins from each study (n=754), the full list and code can be found on github. The column "Protein.Group" represent Uniprot identifiers. Abbreviations: CSF;cerebrospinal fluid.

Genes	Protein.Group	This study, CSF	This study, plasma	Angel, CSF	Gegotek, serum	Nilsson, CSF	Nilsson, Serum	Fredriksson, Plasma
**A1BG**	**P04217**	P04217		P04217				
**A2M**	**P01023**	P01023		P01023				
**ACTB**	**P60709**	P60709	P60709					
**ADAM22**	**Q9P0K1**	Q9P0K1		Q9P0K1				
**AGT**	**P01019**	P01019		P01019	P01019			
**AHSG**	**P02765**	P02765	P02765					
**APCS**	**P02743**	P02743	P02743					
**APLP1**	**P51693**	P51693		P51693				
**APOA4**	**P06727**	P06727	P06727					
**APOC1**	**P02654**	P02654	P02654					
**APOC3**	**P02656**	P02656	P02656					
**APOD**	**P05090**	P05090	P05090					
**APOE**	**P02649**	P02649	P02649					
**APOM**	**O95445**	O95445	O95445					
**APP**	**P05067**	P05067		P05067				
**ATRN**	**O75882**	O75882	O75882					
**AZGP1**	**P25311**	P25311		P25311				
**B2M**	**P61769**	P61769	P61769					
**BCAN**	**Q96GW7**	Q96GW7		Q96GW7		Q96GW7	Q96GW7	
**C1QB**	**P02746**	P02746	P02746	P02746				
**C1QC**	**P02747**	P02747	P02747	P02747				
**C1R**	**P00736**	P00736		P00736				
**C1RL**	**Q9NZP8**	Q9NZP8	Q9NZP8					
**C2**	**P06681**	P06681	P06681					
**C3**	**P01024**	P01024	P01024	P01024				
**C4BPA**	**P04003**	P04003	P04003		P04003			
**C5**	**P01031**	P01031	P01031					
**C6**	**P13671**	P13671	P13671					
**C9**	**P02748**	P02748	P02748					
**CADM3**	**Q8N126**	Q8N126					Q8N126	
**CD14**	**P08571**	P08571	P08571					
**CD59**	**P13987**	P13987		P13987				
**CDC5L**	**Q99459**	Q99459			Q99459			
**CFD**	**P00746**	P00746		P00746				

**CFH**	**P08603**	P08603		P08603				
**CHGB**	**P05060**	P05060		P05060				
**CLEC3B**	**P05452**	P05452	P05452					
**CNDP1**	**Q96KN2**	Q96KN2		Q96KN2				
**CNTN1**	**Q12860**	Q12860		Q12860				
**CP**	**P00450**	P00450		P00450				
**CPB2**	**Q96IY4**	Q96IY4	Q96IY4					
**CPE**	**P16870**	P16870		P16870				
**CPM**	**P14384**	P14384				P14384		
**CRP**	**P02741**	P02741	P02741					
**CSF1**	**P09603**	P09603		P09603		P09603	P09603	
**CST3**	**P01034**	P01034		P01034				
**CTSB**	**P07858**	P07858		P07858				
**CTSC**	**P53634**	P53634				P53634		
**CTSD**	**P07339**	P07339		P07339				
**CTSS**	**P25774**	P25774				P25774	P25774	
**CYCS**	**P99999**	P99999			P99999			
**ECM1**	**Q16610**		Q16610	Q16610				
**F2**	**P00734**	P00734	P00734					
**FBLN1**	**B1AHL2**	B1AHL2		B1AHL2				
**FCGBP**	**Q9Y6R7**	Q9Y6R7		Q9Y6R7				
**FETUB**	**Q9UGM5**	Q9UGM5	Q9UGM5					
**FGA**	**P02671**	P02671	P02671	P02671				
**FGB**	**P02675**	P02675	P02675	P02675				
**FGG**	**P02679**	P02679	P02679	P02679				
**FN1**	**P02751**	P02751	P02751					
**GPLD1**	**P80108**	P80108	P80108					
**GSN**	**P06396**		P06396	P06396				
**HP**	**P00738**		P00738	P00738				
**HPR**	**P00739**	P00739		P00739				
**HPX**	**P02790**	P02790	P02790					
**HRG**	**P04196**	P04196	P04196	P04196	P04196			
**IGF2**	**P01344**	P01344		P01344				
**IGFALS**	**P35858**	P35858	P35858					
**IGFBP6**	**P24592**	P24592	P24592	P24592				
**IGHG1**	**P01857**	P01857	P01857	P01857				
**IGHG3**	**A0A9H3ZR93**	A0A9H3ZR93		A0A9H3ZR93				
**IGHG4**	**P01861**	P01861	P01861	P01861				
**IGHV1-18**	**A0A0C4DH31**	A0A0C4DH31	A0A0C4DH31					
**IGHV3-15**	**A0A0B4J1V0**	A0A0B4J1V0	A0A0B4J1V0					
**IGHV3-38**	**A0A0C4DH36**	A0A0C4DH36	A0A0C4DH36					
**IGHV3-7**	**P01780**	P01780	P01780					
**IGHV3-74**	**A0A0B4J1X5**	A0A0B4J1X5	A0A0B4J1X5					
**IGKC**	**P01834**	P01834	P01834	P01834				
**IGKV1-16**	**P04430**	P04430	P04430					
**IGKV1-27**	**A0A075B6S5**	A0A075B6S5	A0A075B6S5					
**IGKV1-5**	**P01602**	P01602	P01602	P01602	P01602			
**IGKV2-28**	**A0A075B6P5**	A0A075B6P5	A0A075B6P5					
**IGKV2-40**	**A0A087WW87**	A0A087WW87	A0A087WW87					
**IGKV3-11**	**P04433**	P04433	P04433					
**IGKV3-20**	**P01619**	P01619	P01619	P01619				
**IGKV3-7**	**A0A075B6H7**	A0A075B6H7	A0A075B6H7					
**IGKV3D-15**	**A0A087WSY6**	A0A087WSY6		A0A087WSY6				
**IGKV3D-20**	**A0A0C4DH25**	A0A0C4DH25	A0A0C4DH25					
**IGKV4-1**	**P06312**	P06312	P06312	P06312				
**IGKV6-21**	**A0A0C4DH24**	A0A0C4DH24	A0A0C4DH24					
**IGLC7**	**A0M8Q6**	A0M8Q6	A0M8Q6					
**ITIH1**	**P19827**	P19827		P19827	P19827			
**ITIH2**	**P19823**	P19823		P19823	P19823			
**ITIH4**	**Q14624**	Q14624	Q14624					
**KLK6**	**Q92876**	Q92876		Q92876				
**KLKB1**	**P03952**	P03952	P03952					
**KNG1**	**P01042**	P01042	P01042					
**LSAMP**	**Q13449**	Q13449		Q13449				
**LUM**	**P51884**	P51884	P51884		P51884			
**LYNX1**	**P0DP58**	P0DP58		P0DP58				
**LYZ**	**P61626**	P61626		P61626				
**NCAM1**	**P13591**	P13591		P13591				
	**A0A0D9SF30**	A0A0D9SF30		A0A0D9SF30				
**NCAN**	**O14594**	O14594				O14594	O14594	
**NEGR1**	**Q7Z3B1**	Q7Z3B1		Q7Z3B1				
**NEO1**	**Q92859**	Q92859		Q92859				
**NRCAM**	**Q92823**	Q92823				Q92823		
**NRXN2**	**Q9P2S2**	Q9P2S2		Q9P2S2				
**OGN**	**P20774**	P20774		P20774				
**OMG**	**P23515**	P23515		P23515				
**PFN1**	**P07737**	P07737	P07737					
**PLG**	**P00747**	P00747	P00747					
**PLXNB1**	**O43157**	O43157				O43157		
**PROC**	**P04070**	P04070	P04070					

**PROS1**	**P07225**	P07225	P07225	P07225				
**PTPRZ1**	**P23471**	P23471		P23471				
**PZP**	**P20742**		P20742	P20742				
**RBP4**	**P02753**	P02753	P02753	P02753	P02753			
**RGMA**	**Q96B86**	Q96B86				Q96B86		
**S100A8**	**P05109**	P05109	P05109					
**S100A9**	**P06702**	P06702	P06702					
**SCG2**	**P13521**	P13521		P13521				
**SCG3**	**Q8WXD2**	Q8WXD2		Q8WXD2				
**SCG5**	**P05408**	P05408		P05408				
**SELL**	**P14151**	P14151	P14151					
**SERPINA1**	**P01009**	P01009		P01009				
**SERPINA3**	**P01011**		P01011	P01011				
**SERPINA4**	**P29622**	P29622	P29622					
**SERPINA5**	**P05154**	P05154	P05154					
**SERPINA6**	**P08185**		P08185	P08185				
**SERPIND1**	**P05546**	P05546	P05546					
**SERPINF2**	**P08697**	P08697		P08697				
**SERPING1**	**P05155**		P05155	P05155				
**SOD1**	**P00441**	P00441		P00441				
**SPARCL1**	**Q14515**	Q14515		Q14515				
**SPP1**	**P10451**	P10451		P10451				
**TF**	**P02787**		P02787	P02787				
**THY1**	**P04216**	P04216		P04216		P04216		
**TIMP2**	**P16035**	P16035		P16035				
**TNFRSF21**	**O75509**	O75509				O75509		
**TTR**	**P02766**	P02766	P02766					
**TXNDC5**	**Q8NBS9**	Q8NBS9			Q8NBS9			
**VTN**	**P04004**	P04004	P04004	P04004				
**VWC2**	**Q2TAL6**	Q2TAL6					Q2TAL6	
